# Striking Visualization of Diffuse Congenital Nesidioblastosis on Ga-68 DOTATATE PET/CT

**DOI:** 10.4274/mirt.galenos.2018.38039

**Published:** 2019-06-24

**Authors:** Fevziye Canbaz, Murat Aydın, Bilge Can Meydan, Meltem Ceyhan Bilgici, Ender Arıtürk

**Affiliations:** 1 Ondokuz Mayıs University Hospital, Department of Nuclear Medicine, Samsun, Turkey; 2 Ondokuz Mayıs University Hospital, Department of Pediatric Endocrinology, Samsun, Turkey; 3 Ondokuz Mayıs University Hospital, Department of Pathology, Samsun, Turkey; 4 Ondokuz Mayıs University Hospital, Department of Pediatric Radiology, Samsun, Turkey; 5 Ondokuz Mayıs University Hospital, Department of Pediatric Surgery, Samsun, Turkey

**Keywords:** Nesidioblastosis, hyperinsulinemic hypoglycemia, differential diagnosis, Ga-68 DOTATATE PET/CT

## Abstract

“Nesidioblastosis”, later renamed as “persistent hyperinsulinemic hypoglycemia of infancy” presents as either focal or diffuse neo-differentiation of pancreatic Langerhans islet cells from the ductal epithelium. Differentiation of focal disease from diffuse involvement is crucial for optimal disease management. The current methods used to differentiate the two forms pre-operatively are invasive techniques. The definite role of imaging modalities to differentiate diffuse versus focal form has not yet been proven. Herein, we report a 15 day-old infant having diffuse nesidioblastosis, successfully demonstrated by Ga-68 DOTATATE positron emission tomography/computed tomography imaging that was histopathologically confirmed.

## Abstract (tr)

Pankreasın Langerhans ada hücrelerinin duktus epitelinden yeniden farklılaşması olan “nesidioblastosis”, yeni adlandırılmasıyla “yenidoğanın inatçı hiperinsülinemik hipoglisemisi”, fokal ya da diffüz olarak iki şekilde görülebilir. Fokal formun diffüz olandan ayırt edilmesi hastalığın doğru yönetimi için son derece önemlidir. Bu iki formun cerrahi öncesi ayrımında kullanılan güncel metodlar invaziftir. Görüntülemenin diffüz formu fokal olandan ayırt etmedeki rolü tam olarak ortaya konamamıştır. Burada, Ga-68 DOTATATE pozitron emisyon tomografi/bilgisayarlı tomografi görüntüleme ile başarılı bir şekilde gösterilen ve histopatolojik olarak da doğrulanan diffüz nesidioblastosis hastası 15 günlük bir infant olgusunu sunduk.

## Figures and Tables

**Figure 1:**
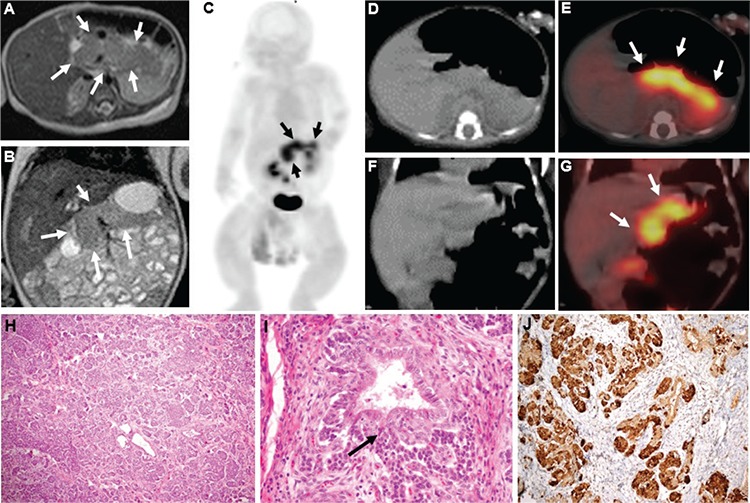
A 15-day-old infant presented with hyperinsulinemic hypoglycemia suffering from hypotonia, apnea and poor feeding. On physical examination, the abdomen was distended. The laboratory investigations revealed hypoglycemia (12 mg/dL, normal range: 70-110 mg/dL) and hyperinsulinemia with serum insulin levels of 55 IU/mL (normal range: 2,42-13 IU/mL). The baby was administered intravenous glucose infusion up to 20 mg/kg/min and oral feeding was supported with glucose to maintain euglycemic state. After intravenous diazoxide (15 mg/kg/day) and octreotide therapy (30- 45 mcg/kg/day), no sufficient response could be obtained. Axial (A) and coronal (B) T2-weighted magnetic resonance images demonstrated diffuse enlargement of the pancreas with normal parenchymal signal without any focal lesion. Ga-68 DOTATATE positron emission tomography/computed tomography (PET/CT) imaging [maximum intensity projection anterior (C), axial CT (D), fused PET/CT (E), coronal CT (F) and fused PET/CT (G)] showed diffusely increased tracer uptake (SUV_max_: 4,84; 3,99 and 3,99 for head, corpus and tail of the pancreas, respectively) in the entire enlarged pancreas (arrows) (with physiological radiotracer distribution throughout the rest of the body) suggesting a diffuse variant of nesidioblastosis. Due to the persistent hyperinsulinemic hypoglycemia and considering the findings in the Ga-68 DOTATATE PET/CT somatostatin receptor imaging, the patient underwent near total pancreatectomy. Histopathologic findings confirmed the diagnosis of diffuse nesidioblastosis, demonstrating diffuse enlargement of pancreatic lobules composed of solid endocrine cell clusters without a tumor. The disarray of lobular architecture with diffuse and irregular hyperplasia of endocrine cells (H: H&E, x100), the continuity between the duct epithelium and endocrine cells (the ductulo-insular complex, arrow) (I: H&E, x200) and immunohistochemistry with chromogranin A staining endocrine cells (J: x200) are demonstrated. Congenital hyperinsulinism is the most common cause of persistent hypoglycemia in infancy, existing in two forms of either focal or a diffuse adenomatous hyperplasia of insulin secretion in the pancreas. The pre-operative differentiation of these two conditions is crucial for disease management (1,2). Focal type can be treated by selective surgical resection in contrast to the diffuse form which requires near total pancreatectomy when resistant to medical treatment (3). No clinical or biological features are typical in determining disease type in affected infants. The current methods used for pre-operative differentiation are invasive techniques and do not always provide differential diagnosis (4,5,6). The definite role of imaging modalities to differentiate diffuse versus focal form has not yet been proven. F-18-fluoro-dihydroxyphenylalanine PET scan has been used in case of hyperinsulinemia with a reported accuracy of 96% in diagnosing focal or diffuse disease, and of 100% in localizing the focal lesion (3,7). To the best of our knowledge, only few case reports have been published regarding the role of somatostatin receptor imaging to distinguish focal disease from diffuse involvement, where Ga-68 DOTATATE PET scan had been applied successfully in one case and Ga-68 DOTATOC PET scan has been reported to have limited success in another report (1,8). The presented case is evident with an enlarged pancreas showing diffuse increased Ga-68 DOTATATE uptake and indicates somatostatin receptor imaging as a valuable option to guide the type of pancreatectomy in patients with persistent hyperinsulinemic hypoglycemia
